# Supplementary material for: Hair Growth Effect and the Mechanisms of Rosa rugosa Extract in DHT-Induced Alopecia Mice Model
Source: Int J Mol Sci. 2024 Oct 22;25(21):11362. doi: 10.3390/ijms252111362 (PMC11545796; doi:10.3390/ijms252111362)
Supplement: Supplementary file 1 [file ijms-25-11362-s001.zip › ijms-3218568-supplementary.pdf]

Supplementary Table S1. Analysis condition of UPLC

| Parameter | Condition | |
|---|---|---|
| UPLC system | Waters ACQUITY UPLC I-Class Plus System | |
| Column | ACQUITY UPLC® BET C18 1.7 μm(2.1 × 100 mm) | |
| Mobile phase | A | LC-MS grade water<br>(0.1% formic acid) |
| | B | LC-MS grade acetonitrile<br>(0.1% formic acid) |
| | Gradient | 0 min: A/B = 98/2<br>10 min: A/B = 89/11<br>12 min: A/B = 89/11<br>14 min: A/B = 80/20<br>20 min: A/B = 77/23<br>35 min: A/B = 50/50<br>38.5 min: A/B =10/90<br>39 min: A/B = 0/100<br>40 min: A/B = 0/100 |
| Flow rate | 0.2 mL/min | |
| Injection volume | 1 μL | |
| Sample concentration | 2 mg/mL (in LC-MS grade water) | |
| Column oven Temp. | 30 ℃ | |
| Run time | 40 min | |
| Wavelength | PDA | |

Supplementary Table S2. Analysis condition of Q-TOF-MS

| Parameter | Condition |
|---|---|
| Q-TOF-MS system | Waters Xevo G2-XS Q-TOF–MS |
| Method type / Source | $MS^e$/ ESI |
| Polarity / Analysis mode | Negative / Resolution |
| Source Temp. | 150 ℃ |
| Desolvation Temp. | 350 ℃ |
| Calibration | 0.5 mM Sodium formate |
| Lock Spray | 200 pg/mL Leucine enkephaline |

Supplementary Figure S1. Chromatogram data of UPLC-Q-TOF-MS in RWE

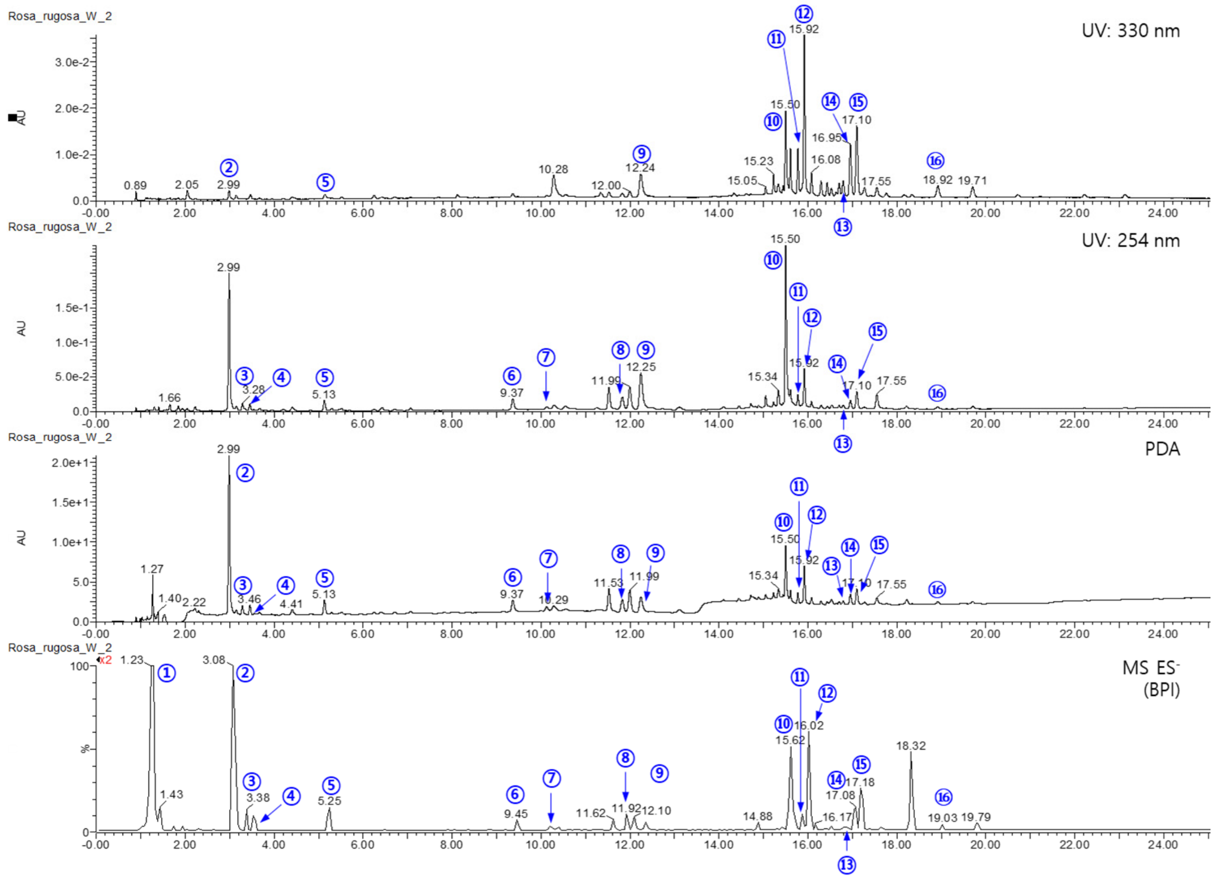

Supplementary Table S3. UPLC-Q-TOF-MS data in RWE

| **No.** | **RT (min)[1)]** | **$[M - H]^-$** | **Error (ppm)** | **Molecular Formula** | **Putative structure** |
|---|---|---|---|---|---|
| **1** | 1.23 | 191.0575 | 9.9 | $C_7H_{12}O_6$ | Quinic acid |
| **2** | 3.08 | 169.0150 | 7.7 | $C_7H_6O_5$ | Gallic acid |
| **3** | 3.38 | 343.0674 | 2.6 | $C_{14}H_{16}O_{10}$ | Galloylquinic acid |
| **4** | 3.53 | 483.0791 | 3.3 | $C_{20}H_{20}O_{14}$ | Di-O-galloyl glucose |
| **5** | 5.25 | 483.0791 | 3.3 | $C_{20}H_{20}O_{14}$ | Di-O-galloyl glucose |
| **6** | 9.45 | 785.0849 | 0.9 | $C_{41}H_{22}O_{17}$ | Ellagitannin (Digalloyl-HHDP-glucose) |
| **7** | 10.21 | 635.0899 | 2.4 | $C_{27}H_{24}O_{18}$ | Tri-O-galloyl glucose |
| **8** | 11.92 | 785.0849 | 0.9 | $C_{41}H_{22}O_{17}$ | Ellagitannin (Digalloyl-HHDP-glucose) |
| **9** | 12.36 | 469.0047 | 0.9 | $C_{21}H_{10}O_{13}$ | Flavogallonic acid |
| **10** | 15.62 | 300.9986 | 0.7 | $C_{14}H_6O_8$ | Ellagic acid |
| **11** | 15.87 | 463.0902 | 5.6 | $C_{21}H_{20}O_{12}$ | Hyperoside |
| **12** | 16.02 | 463.0902 | 5.6 | $C_{21}H_{20}O_{12}$ | Isoquercitrin |
| **13** | 16.88 | 433.0770 | -0.2 | $C_{20}H_{18}O_{11}$ | Quercetin-3-O-arabinoside |
| **14** | 17.08 | 447.0915 | -2.7 | $C_{21}H_{20}O_{11}$ | Astragalin |
| **15** | 17.18 | 447.0915 | -2.7 | $C_{21}H_{20}O_{11}$ | Quercitrin |

| | | | | | |
|---|---|---|---|---|---|
| **16** | 19.03 | 431.0999 | 4.9 | $C_{21}H_{20}O_{10}$ | Kaempferol-3-O-rhamnoside |

1) Retention time, RT

Supplementary Figure S2.

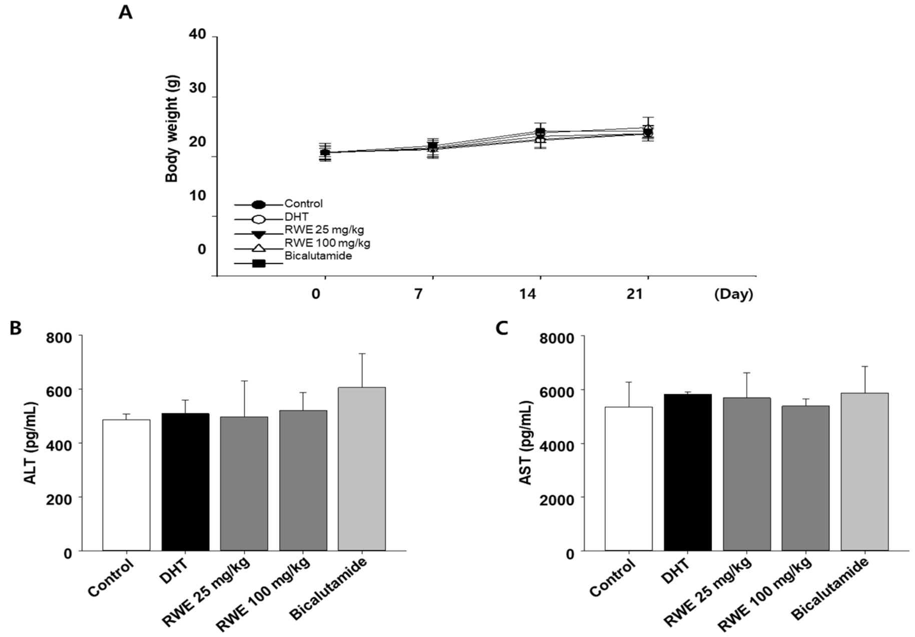


**Supplementary Figure S2.** Hepatotoxicity Indicators of RWE in DHT-Induced Mice. Change of Body weight in mice over the experimental period (A). AST and ALT levels to assess hepatotoxicity of treatments in Serum (B). There was no significant difference in the data. The data were evaluated using one-way ANOVA, with subsequent analysis performed using Tukey's multiple comparison test.
